# Transabdominal ultrasound for the characterization and follow-up of cystic pancreatic lesions

**DOI:** 10.1038/s41598-025-07136-w

**Published:** 2025-06-20

**Authors:** Julian Seelig, Maria Heni, Max Seitzinger, Kien Vu Trung, Jürgen Feisthammel, Marcus Hollenbach, Robert Henker, Albrecht Hoffmeister, Jonas Rosendahl, Valentin Blank, Thomas Karlas

**Affiliations:** 1 Department of Medicine II, Division of Gastroenterology, Leipzig University Medical Center, Liebigstrasse 20, 04103 Leipzig, Germany; 2 Division of Interdisciplinary Ultrasound, Department of Internal Medicine I, University Hospital Halle, Ernst-Grube-Straße 40, 06120 Halle(Saale), Germany; 3 Department of Gastroenterology, Metabolism, Clinical Infectiology and Gastrointestinal Oncology, University Hospital Marburg, 35043 Baldingerstraße, Marburg, Germany; 4 Department of Gastroenterology, Erzgebirgsklinikum Zschopau, Alte Marienberger Straße 52, 09405 Zschopau, Germany

**Keywords:** Cystic pancreatic lesions, Endosonography, Pancreas, Transabdominal ultrasound, Pancreatic disease, Pancreatic cancer

## Abstract

Cystic pancreatic lesions (CPL) pose a diagnostic challenge due to their morphological diversity and malignant potential. Given the limited study data, transabdominal ultrasound (TAUS) is currently not established for either primary diagnostics or CPL monitoring. This study compared the diagnostic accuracy of TAUS in the assessment of CPL to that of the reference method, endoscopic ultrasound (EUS), to identify patient subgroups suitable for TAUS monitoring. In a monocentric, retrospective analysis, patients with CPL who underwent EUS and TAUS within six months from 01/2016 to 06/2022 were included. Univariate and multiple logistic regression analyses were used to identify determinants for the detection of CPL via TAUS. Cross-method morphological assessments were analysed, and a patient-specific algorithm for selecting the appropriate monitoring method was developed. Among 105 patients, CPL were detected by both EUS and TAUS in 90 patients (86%). Patients with “TAUS negative” CPL (*n* = 15) exhibited greater body mass indices (BMI, *p* = 0.002) and smaller CPL diameters (*p* = 0.043). The final multivariate model (BMI, age, CPL diameter) yielded an 85% accuracy in predicting CPL detectability by TAUS. TAUS could be a cost-effective and patient-friendly imaging method for the surveillance of CPL in selected patients.

**Supplementary Information:**

The online version contains supplementary material available at 10.1038/s41598-025-07136-w.

## Introduction

Cystic pancreatic lesions (CPL) comprise a wide spectrum of both neoplastic and nonneoplastic processes characterized by one or multiple fluid-filled cavities^1^. CPL are often asymptomatic, and the majority is detected as an incidental finding during routine imaging procedures^2,3^. Comprehensive diagnostic assessment is required for lesions larger than 5 mm in diameter to determine the prognosis of the CPL due to the divergent malignant potential of the various entities. While many CPL remain benign over time, some, such as main-duct intraductal papillary mucinous neoplasms (IPMNs) and mucinous cystic neoplasms (MCNs), have a significant risk of progressing to pancreatic cancer^4^.

For the identification of CPL with the potential for malignant transformation, well-defined image morphological predictors, so-called “worrisome features”, have been identified. These included lesion size, wall characteristics and affection of the pancreatic duct^5^. The progression of the number of worrisome features leads to a stepwise increase in the risk of developing malignant processes from < 25% to almost 100%^6^.

Consequently, a detailed evaluation of imaging characteristics is crucial for effective risk stratification, in addition to considering patient-specific parameters such as age, sex and comorbidities^7^. Patients with CPL displaying worrisome features often require surgery due to the high risk of malignant transformation. In contrast, low-risk patients are commonly assigned to observational programs to identify morphologic changes and potential size progression.

However, the optimal follow-up strategy for low-risk CPL lesions is a subject of ongoing debate^8–10^. Various international guidelines propose different follow-up intervals^11^and the termination of surveillance in patients with stable findings remains an unsolved problem^12^. Thus, the increasing incidence of incidental CPL diagnosis by modern imaging techniques^13^ results in a challenging and resource-consuming need for the long-term management of affected patients. A particular issue is the exclusive recommendation of endoscopic ultrasound (EUS) and magnetic resonance imaging (MRI) as appropriate follow-up imaging examinations in CPL patients^11^. Both methods are accurate for differentiating between low- and high-risk CPL but require considerable human and technical resources and have limited capacities for nationwide surveillance programs.

Transabdominal ultrasound (TAUS) is the first-line imaging method for a variety of abdominal medical conditions and is a frequent source of incidental CPL detection. It is widely available, noninvasive, and potentially a cost-effective alternative to EUS and MRI, especially for the follow-up management of low-risk CPL. TAUS may achieve an accurate depiction of CPL in the majority of patients if qualified and experienced examiners apply standardized protocols^14^.

However, although TAUS is frequently used in clinical practice, this method has not yet been endorsed by official guideline recommendations because only limited evidence is available; hence, concerns regarding the sensitivity of this method for the detection of CPL and an adequate description of worrisome features persist^15^. Few previously published retrospective studies revealed high cross-method reliability between TAUS and EUS^16^but a relevant proportion of the included patients displayed insufficient agreement in the description of morphological irregularities and the subclassification of CPL^17^. In the present study, we aimed to develop a clinical decision model that may enable the identification of patient subgroups potentially eligible for accurate TAUS monitoring in future prospective studies.

## Results

### Study cohort and patient characteristics

**Fig. 1:**
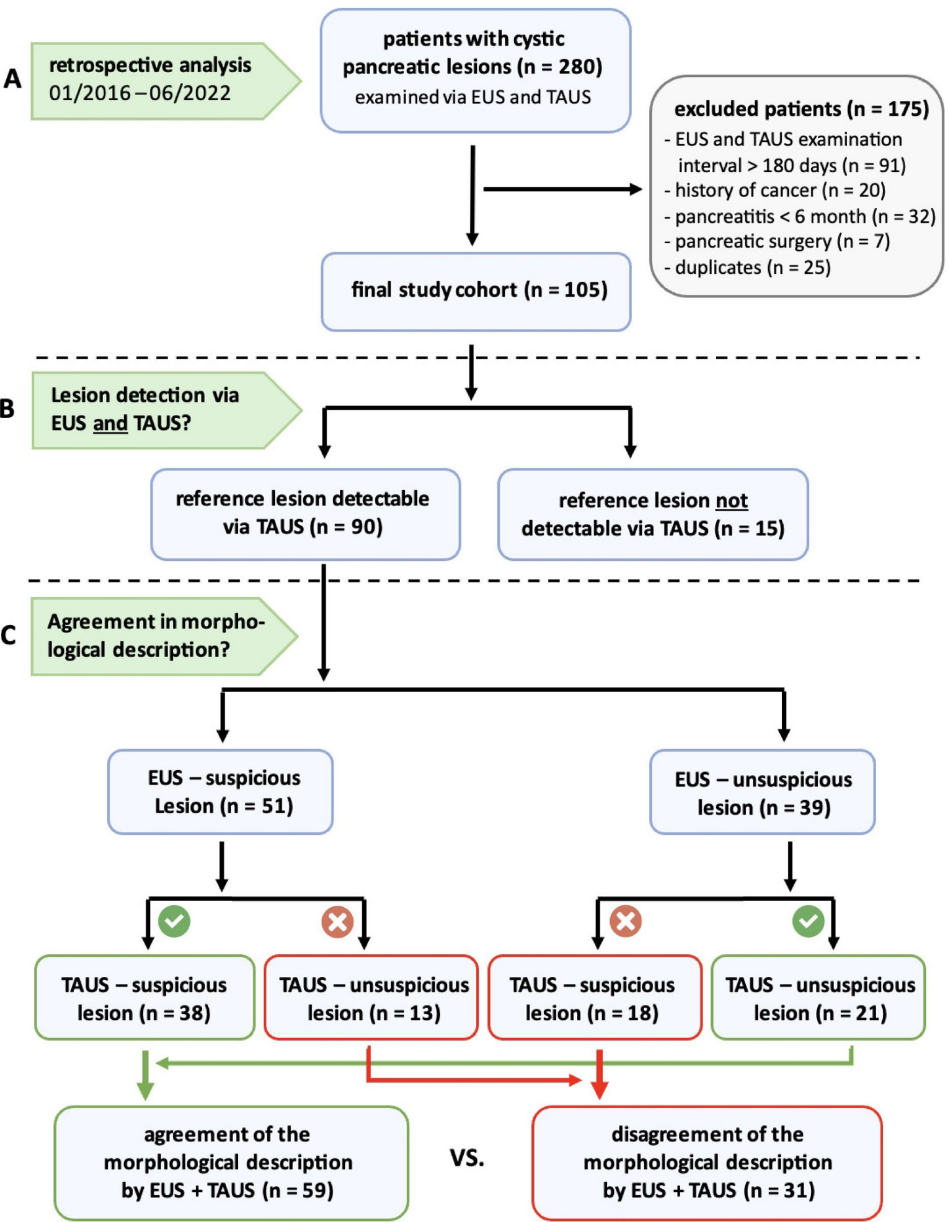
Flowchart of the study procedure. A retrospective analysis was performed at Leipzig University Hospital to identify all patients with cystic pancreatic lesions (CPL) who underwent endoscopic ultrasound (EUS) and transabdominal ultrasound (TAUS). The final study population (**A**) was analysed for cross-method detectability of the reference lesions using EUS and TAUS (**A**). Potential determinants of TAUS detectability were investigated by subgrouping lesions into “detectable via TAUS” and “not detectable via TAUS” (**B**). Reference lesions detected by both methods were evaluated for their cross-method agreement in terms of morphological characterization and risk stratification (**C**).

A total of 280 patients with CPL were identified in the predefined period from 01/2016 to 06/2022. After applying the inclusion and exclusion criteria, 105 patients/CPL were included in the data analysis (Fig. 1). In 83 of 105 patients with CPL (79%), TAUS was the initial imaging method, followed by EUS after an average interval of 30 days. In contrast, EUS served as the index examination in 22 of 105 patients with CPL (21%), with subsequent TAUS obtained after an average of 73 days. Patient characteristics (age, sex, and BMI) of the final study cohort as well as specifications of the cystic reference lesion (localization and size determined via EUS) are shown in Table 1.

**Table 1:** Patient characteristics of the study cohort stratified by TAUS detectability.

Parameter	total cohort (*n* = 105)	CPL detectable by EUS and TAUS (*n* = 90)	CPL detectable only by EUS (*n* = 15)	*p* value
BMI (kg/m²)				*p* = 0.002
median [IQR]	24.8 [22.0, 28.3]	24.2 [21.8, 27.9]	27.9 [25.9, 30.6]	
age (years)				*p* = 0.054
median [IQR]	69.0 [58.0, 77.0]	67.0 [57.0, 76.0]	76.0 [65.0, 78.0]	
sex				*p* = 0.406
female (%)	66 (62.9)	58 (64.4)	8 (53.3)	
EUS diameter (mm)				*p* = 0.043
median [IQR]*	12.0 [7.0, 20.0]	12.0 [8.0, 20.0]	8.0 [5.0, 16.2]	
< 1 cm (%)	35 (35.7)	26 (30.9)	9 (86.6)	
≥ 1 cm (%)	63 (64.3)	58 (69.1)	5 (6.7)	
localization				*p* = 0.957
caput (%)	52 (49.5)	44 (48.8)	8 (53.3)	
corpus (%)	43 (40.9)	37 (41.1)	6 (40.0)	
cauda (%)	5 (4.8)	4 (4.5)	1 (6.7)	
multiple (%)	5 (4.8)	5 (5.6)	0 (0.0)	

Abbreviations: TAUS - transabdominal ultrasound; EUS - endoscopic ultrasound; IQR - interquartile range. To analyse group differences in BMI, age, and tumour diameter (determined via EUS), the Mann‒Whitney U test was used. Fisher´s exact test was performed to analyse the differences in sex distribution and lesion location. *Missing data from seven patients due to technical limitations in the sizing of too-extended lesions and due to statistical outlier correction.

Based on all available imaging and clinical data, the spectrum of CPL entities comprised intraductal papillary mucinous neoplasia (IPMN; *n* = 31; 23 branch duct, 6 main duct and 2 mixed-type IPMN cases, respectively), mucinous cystic neoplasia (MCN; *n* = 1), serous cystic neoplasia (SCN; *n* = 7), malignant cystic lesions (*n* = 3), pseudocysts (*n* = 14), cystic lesions not further classified (in the absence of clinical consequences) without worrisome features (*n* = 49). The classification was based on imaging in 87 CPL, whereas cytology or histopathology was available in 18 cases.

### Cross-method identification of the cystic reference lesions

In the final study cohort (*n* = 105), in 90 patients (85.7%) CPL could be detected by both EUS and TAUS (classified as “TAUS detectable”), while in 15 patients (14.3%) CPL were only visible via EUS (classified as “TAUS not detectable”). In 13/15 of the latter cases, TAUS was performed prior to EUS.

Patients with “TAUS not detectable” CPL exhibited a significantly greater BMI (*p* = 0.002) and a smaller diameter of the CPL (*p* = 0.043, measured via EUS) than patients with detectable CPL via TAUS. In addition, the patients with CPL detectable via TAUS tended to be younger (67.0 vs. 76.0 years; *p* = 0.054). The sex distribution and localization of CPL were comparable between the groups. Consequently, CPL detection rates via TAUS were comparable for the different CPL localizations, caput (*n* = 52; sensitivity 86%), corpus (*n* = 43, sensitivity 86%), cauda (*n* = 5, sensitivity 80%) and for multiple CPL (*n* = 5, sensitivity 100%). Details are given in Table 1 and **supplemental Fig. 1**.

### Evaluation of patient- and lesion-specific data in predicting the detectability of cystic pancreatic lesions via TAUS

**Fig. 2:**
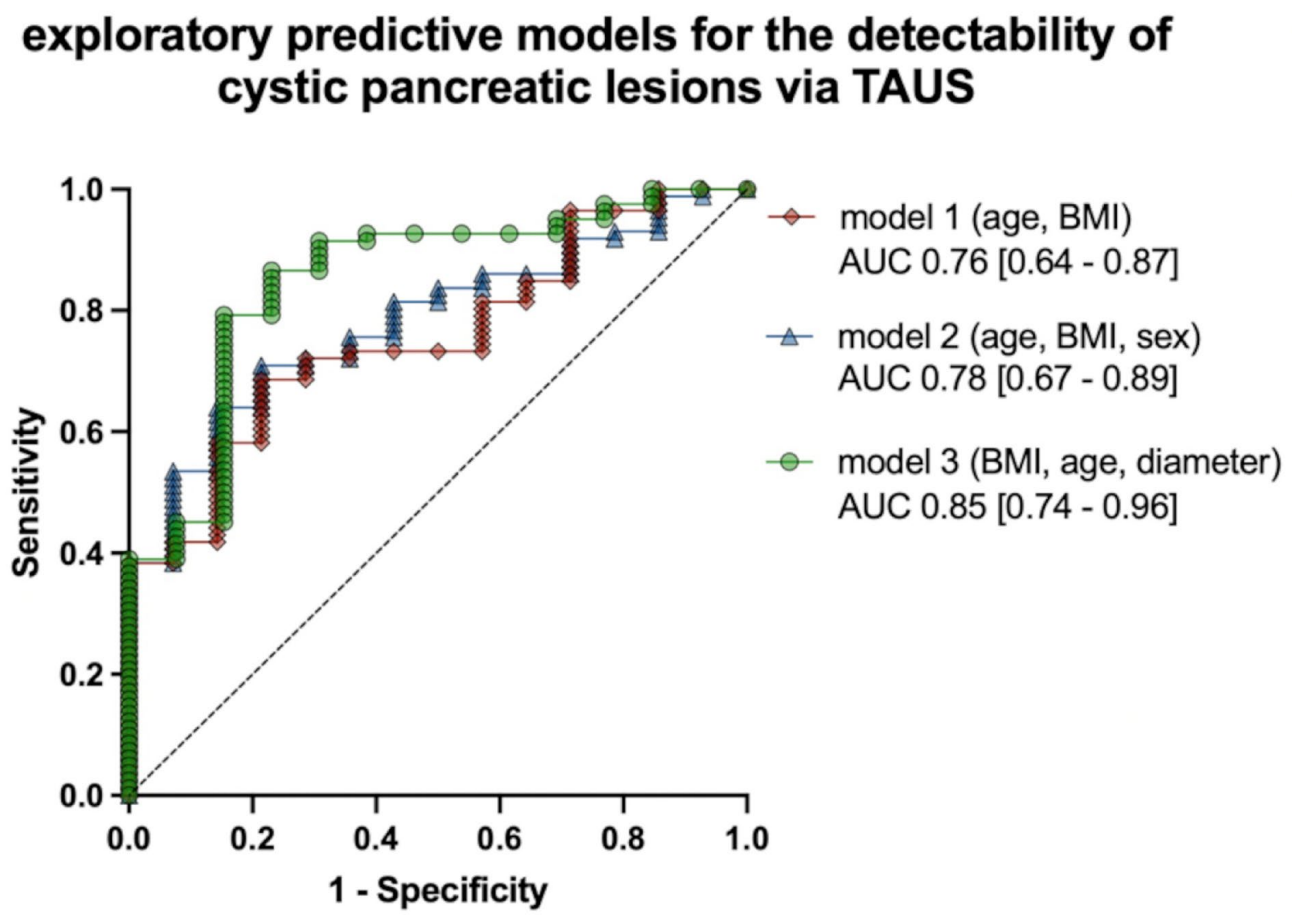
Multiple logistic regression analysis of combined patient characteristics and lesion-specific data for predicting the detectability of pancreatic cystic lesions (CPL) by transcutaneous ultrasound (TAUS). Based on the combination of different parameters, predictive models (model 1 and model 2) for the detection of CPL by TAUS can be generated with moderate precision (AUC 0.76 and 0.78). Assessing the different models with stepwise AIC selection, the model based on BMI, patient age and EUS diameter (model 3) showed the highest AUC (0.85) of all combinations.

Multiple logistic regression analyses were performed to explore the predictive value of patient- and lesion-specific data in predicting the detectability of the CPL via TAUS. The receiver operating characteristic (ROC) curves of the calculated models considering age and BMI (Model 1) and the combination of age, BMI and sex (Model 2) showed moderate precision in predicting TAUS detectability, with area under the curve (AUC) values of 0.76 and 0.78, respectively. The final model based on BMI, age, and diameter of the CPL (Model 3) outperformed all other combinations of patient and CPL specific parameters (including CPL localization, CPL morphology and morphology of pancreatic duct system e.g. dilatation, caliber changes and CPL connection) applying stepwise AIC selection with the highest AUC of 0.85 (Fig. 2).

### Comparative cross-method diameter analyses

The lesion diameters determined with EUS and TAUS were compared to assess the agreement in size of the CPL between both methods. For the analysis, an outlier adjustment was first carried out to obtain a homogeneous dataset. Subsequent analysis of the adjusted cohort (*n* = 84) revealed a significant difference in intermodal size (*p* = 0.0034), while maintaining a strong correlation (*r* = 0.78, *p* < 0.0001).

**Fig. 3:**
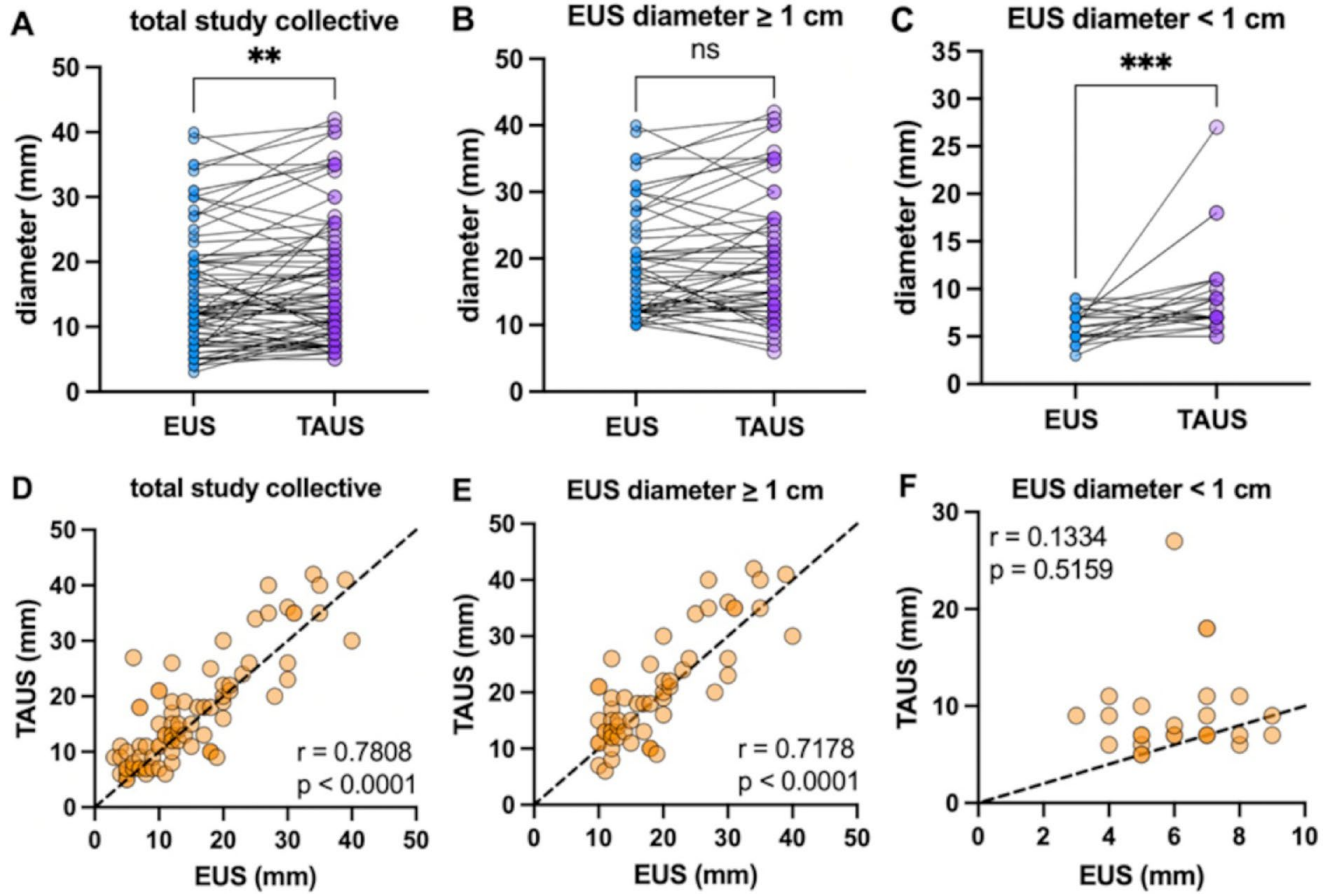
Comparative cross-method diameter analyses of cystic pancreatic lesions (CPL) detected by endoscopic endosonography (EUS) and transabdominal ultrasound (TAUS) (**A-F**). Significant differences in the size of CPL detected via EUS and TAUS were found for the total study population (**A**). The correlation analyses showed highly significant cross-method agreement (**D**). CPL < 1 cm differed highly significantly between EUS and TAUS (**C**) and consequently showed no correlation (**F**). In contrast, CPL ≥ 1 cm (**B**) were not significantly different between the methods (**E**). Correlation analyses were performed using Spearman’s correlation test, and group differences were tested using the Wilcoxon matched pairs signed-rank test. All the statistical tests used an α-level of 0.05, and statistical significance was defined as *p* > 0.05 (ns), *p* < 0.05 (*), *p* < 0.01 (**) or *p* < 0.001 (***).

Dividing the total amount of CPL into two subgroups according to their size revealed differences depending on whether the CPL exceeded the 1 cm limit. There were no significant differences in the sizes of CPL **≥** 1 cm between TAUS and EUS (*p* = 0.1270); consequently, the values were highly significantly correlated (*r* = 0.72, *p* < 0.0001). In contrast, CPL < 1 cm displayed significant differences in size determination between the methods (*p* = 0.0005) and accordingly exhibited no cross-method correlation (*r* = 0.13, *p* = 0.5159). The details are given in Fig. 3.

### Evaluation of cross-method agreement in the morphologic assessment of CPL

**Fig. 4:**
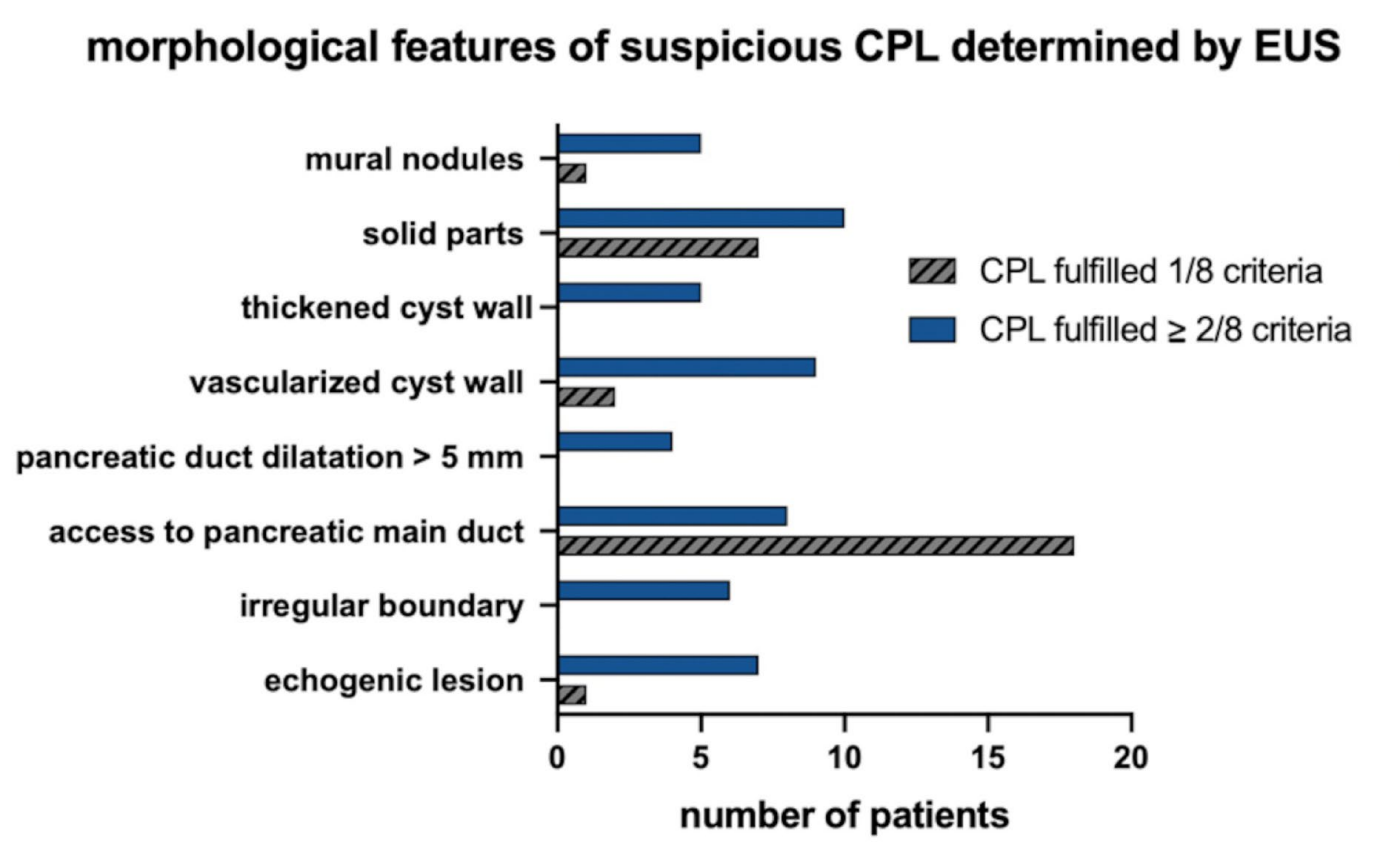
Distribution and frequency of morphologically suspicious features of pancreatic cystic lesions (CPL) detected by endoscopic ultrasound (EUS).

Morphologic descriptions of CPL were available in a total of 90 patients for both modalities (EUS and TAUS). In the EUS reference examination, 51 (56.7%) CPL were classified as morphologically “suspicious” according to the predefined criteria, while 39 (43.3%) CPL were categorized as morphologically “unsuspicious”. Among the 51 suspicious CPL cases, 29 (56.9%) fulfilled 1/8 of the criteria, and 22 (43.1%) met ≥ 2/8 of the criteria (see Fig. 4). In 13 of these cases, invasive diagnostics (fine needle aspiration, surgery or equivalent) were performed, with the detection of two MD-IPMN with low grade dysplasia, one BD-IPMN with low grade dysplasia, three pseudocysts, four SCN, one lymphoepithelial cyst and two cases of adenocarcinoma. In a cross-method comparison, 38 (74.5%) of the 51 “EUS-suspicious” CPL were also classified as suspicious by TAUS. Of the 39 “EUS-unsuspicious” CPL, 21 CPL (53.8%) were also classified as unsuspicious by TAUS.

Focusing on criteria that defined a “suspicious lesion”, 20/29 (69.0%) of the CPL with one suspicous feature at EUS were also classified as suspicious via TAUS. Among CPL that fulfilled ≥ 2/8 of the EUS criteria (*n* = 22), 18/22 (81.8%) were also classified as suspicious via TAUS. Overall, there was agreement in the cross-method morphologic assessment of CPL (suspicious vs. unsuspicious) in the total cohort for 59/90 (65.6%) patients.

To assess parameters impacting cross-method agreement in the morphological description of CPL, the two groups “TAUS and EUS agreement” (*n* = 59) and “TAUS and EUS disagreement” (*n* = 31) were analysed regarding patient characteristics and lesion-specific data. The analysis revealed a comparable distribution of patient sex and CPL location. There were no significant group differences with regard to the BMI or age of the patients, whereas the CPL diameter differed significantly among the groups (*p* = 0.008). Further details are shown in Table 2.

**Table 2:** Comparison of patient characteristics regarding agreement in the morphological description of the cystic pancreatic lesion (CPL) by EUS and TAUS.

parameter	TAUS and EUS agreement (*n* = 59)	TAUS and EUS disagreement (*n* = 31)	*p* value
BMI (kg/m²)			*p* = 0.905
median [IQR]	24.4 [21.7, 28.0]	23.7 [22.2, 27.9]	
age (years)			*p* = 0.289
median [IQR]	69.0 [61.0, 76.0]	61.0 [55.0, 76.0]	
sex			*p* = 0.174
female (%)	35 (60.3)	23 (74.2)	
EUS diameter (mm)			*p* = 0.008
median [IQR]*	15.5 [9.0, 23.8]	10.0 [7.0, 12.0]	
< 1 cm (%)	15 (26.8)	11 (39.3)	
≥ 1 cm (%)	41 (73.2)	17 (60.7)	
localization			*p* = 0.579
caput (%)	29 (49.1)	15 (48.5)	
corpus (%)	24 (40.7)	13 (41.9)	
cauda (%)	3 (5.1)	1 (3.2)	
multiple (%)	3 (5.1)	2 (6.4)	

Abbreviations: TAUS - transabdominal ultrasound; EUS - endoscopic ultrasound; agreement - morphological risk assessment identical by EUS and TAUS; disagreement - morphological risk assessment differing by EUS and TAUS; IQR - interquartile range. To analyse group differences in BMI, age, and tumour diameter (determined via EUS), the Mann‒Whitney U test was used. Fisher´s exact test was performed to analyse the differences in sex distribution and lesion location. *Missing data from six patients due to technical limitations in the sizing of too-extended lesions and due to statistical outlier correction.

### Clinical algorithm for the appropriate follow-up method

**Fig. 5:**
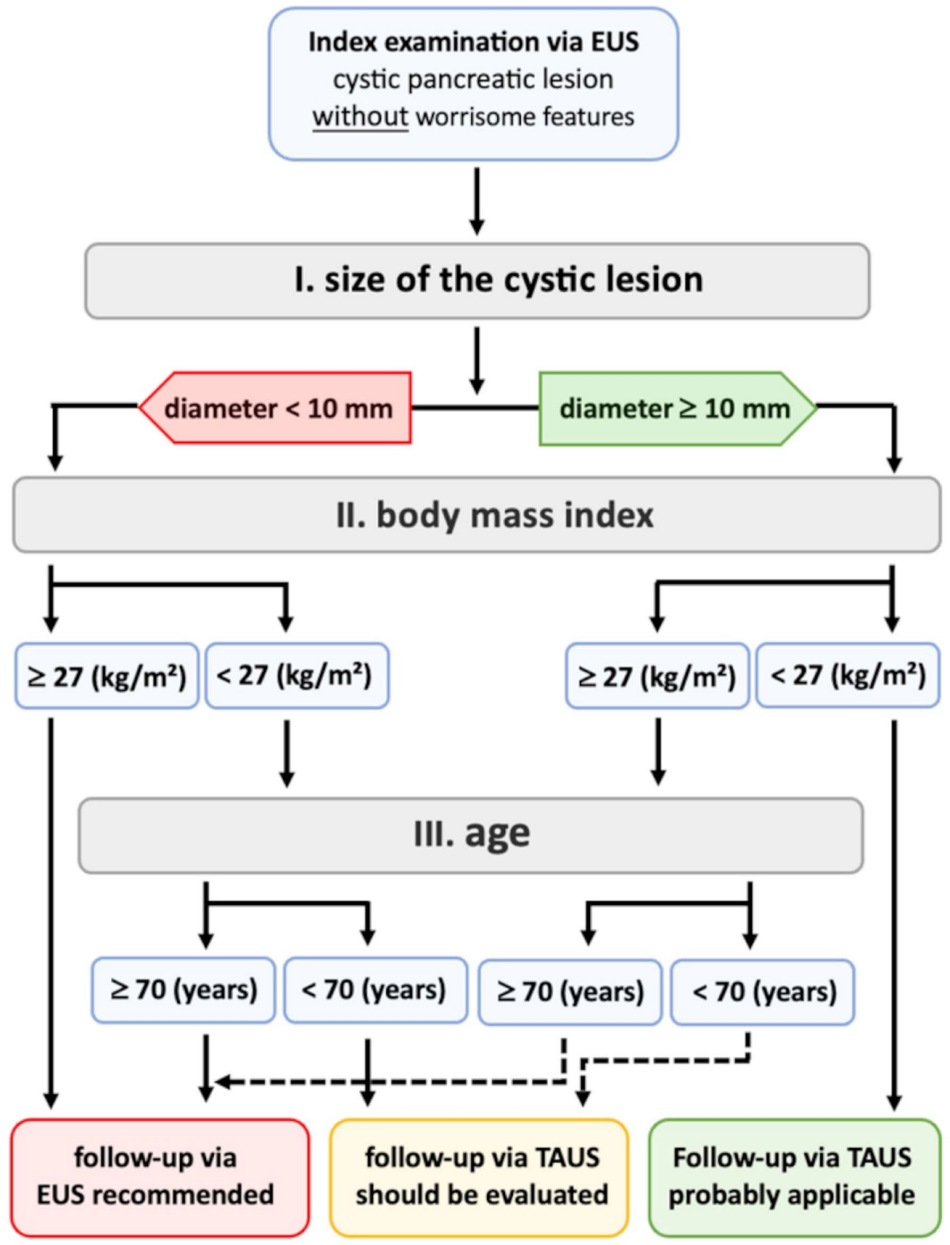
A diagnostic algorithm for evaluating the appropriate follow-up examination method (endoscopic endosonography [EUS] or transabdominal ultrasound [TAUS]) for cystic pancreatic lesions (CPL) without worrisome features is suggested. The diameter of CPL in combination with the patient’s BMI and age determine whether CPL detected via EUS can also be detected via TAUS. Optimal cut-offs for these parameters were calculated under the condition of a univariate detection rate > 90%.

Based on the results of the univariate and multiple logistic regression analyses for the detection of CPL via TAUS, a diagnostic algorithm was developed for referring patients to the appropriate follow-up examination after the index EUS procedure. To determine the decision steps, optimal cut-offs were calculated for the parameters EUS diameter, BMI, and patient age under the condition of a univariate detection rate ≥ 90%. This resulted in a cut-off of 10 mm for the EUS diameter, a cut-off of 27 kg/m² for the BMI and a cut-off of 70 years for patient age. By applying the algorithm to the retrospective dataset of 105 CPL patients, a TAUS detection rate of 97.6% (41/42) was observed for patients with a CPL ≥ 10 mm and a BMI < 27 kg/m². Among the CPL classified as morphologically suspicious via EUS, 85.2% (23/27) of the CPL in these patients could also be identified as suspicious via TAUS. The algorithm is presented in Fig. 5.

## Discussion

Due to demographic changes and continuous technological advances in imaging techniques, the incidence and prevalence of incidentally detected CPL are steadily increasing, requiring personnel and economic resources for surveillance^18,19^. Our results demonstrate that TAUS is a suitable monitoring method for low-risk CPL in selected patient groups. In our cohort, TAUS demonstrated a high CPL detection rate of 85.7% (90/105) compared to that of the reference method EUS. The results align with those of previous retrospective and prospective studies with similar detection rates > 80% for CPL compared to those of MRI or EUS^14,20^.

Patient anthropometry and age as well as lesion size impacted on the accuracy of TAUS for CPL detection. Patients in whom TAUS failed to detect CPL had higher BMI and smaller CPL diameters, which seemed to be especially relevant for younger patients. Consequently, combining these parameters in a multiple logistic regression model enabled the prediction of CPL by TAUS with an accuracy of 85%. Contrary to our initial assumption, further CPL-specific parameters such as CPL localization, CPL morphology and morphology of the pancreatic duct system revealed an inferior predictive value in comparison to the parameters of the final model. However, this could be related to the limited incidence of CPL with morphological irregularities and warrants further evaluation in a larger study population.

The significance of BMI and diameter in CPL detection via TAUS can presumably be attributed to the technical limitations of transcutaneous ultrasound devices and the anatomical location of the pancreas^21^. Previous analyses revealed that an increased BMI and the consecutive presence of a fatty liver resulted in almost 30% less CPL being described via TAUS and tended to be underestimated in size, in comparison to patients without steatotic liver disease^22^. In terms of CPL diameter, a prospective study by Mi Hye Yu et al.. revealed that CPL ≥ 10 mm was more likely to be detected via TAUS than CPL < 10 mm (89.1% vs. 63.6%)^20^, which is consistent with the results of the present study (92.8% vs. 71.4%). This is also reflected in the cross-method analysis of CPL diameters in our cohort, where intermodal agreement depended mainly on lesion size.

The 2024 Kyoto guidelines and further international expert consensus recommend MRI or EUS as the preferred surveillance method for CPL for at least five years^10,23–25^. Otherwise, these guidelines outline the emerging and unresolved economic, personnel and logistical challenges of current monitoring protocols. Despite the retrospective study design and the inherent limitations of lesion characterization, we observed a remarkable detection rate of 74.5% by TAUS for CPL exhibiting morphologically suspicious features. This may not be considered sufficient for immediate adaptation into clinical practice but justifies further prospective evaluation based on standardized protocols with a special focus on worrisome features and high-risk stigmata. In particular, the detection of main pancreatic duct dilatations > 10 mm or the identification of mural nodules or solid parts is essential for appropriate risk assessment^4,10^. As data on the sensitivity of TAUS in detecting these specific morphologies are limited, we currently explicitly refrain from any recommendation to use TAUS as a standard solitary diagnostic method in the initial work-up of CPL. Furthermore, we would like to emphasize that even if CPL is first described via TAUS, at least one complementary EUS and or MRI examination are essential in order to sufficiently rule out relevant morphological irregularities and, especially in the case of corresponding clinical symptoms, coexistent carcinoma of the pancreas.

However, a retrospective analysis by Yu Ji Li et al. showed a high TAUS sensitivity of 82.4% in the detection of pancreatic duct dilatation, whereas only a moderate sensitivity of 30–50% could be described in the detection of solid parts or a thickened cyst wall. The authors concluded that TAUS may be suitable for the follow-up assessment of possible CPL size increases and the evaluation of pancreatic duct dilation^16^. In a retrospective German cohort of 147 patients the diagnostic efficacy of high-resolution ultrasound devices, MRI, CT, and EUS were compared. Larger CPL were well detected with TAUS, whereas changes in the pancreatic duct were least accurately depicted with TAUS compared to the other diagnostic modalities investigated in this cohort^17^. The partially differing study results, especially in the characterization of the pancreatic duct, could be attributed not only to retrospective data evaluation due to its inherently limited data quality but also to the variability of study cohorts from different geopgraphic regions. This underlines the need for prospective and multicenter studies.

To address future challenges, the current study provides a pragmatic scheme based on patient- and lesion-specific determinants of CPL detection, which enables the selection of potentially appropriate patients for CPL monitoring via TAUS (Fig. 5). The application of this algorithm to the retrospective dataset resulted in an improved CPL detection rate with TAUS of 97.6%, with a sensitivity of 85.2% for the detection of morphologically suspicious CPL. This diagnostic scheme still awaits prospective evaluation in an independent cohort but could already provide the rationale for bimodal CPL surveillance in a larger, prospective study to investigate whether TAUS could be a feasible alternative surveillance method for lean patients with low-risk CPL in the future. It must be noted that any prospective attempt in this field requires high-performance ultrasound devices^21,26^must be conducted by experienced and trained examiners according to standardized protocols^27,28^ and must include at least one complementary method (EUS and/or MRI) for simultaneous CPL evaluation.

Our study has several limitations, which are mainly attributed to its retrospective design and moderate sample size, which did not allow stratification by CPL entities. Especially, only 9 out of 31 IPMN CPL showed worrisome features or high risk stigmata. We acknowledge that a better understanding of IPMN imaging features is of high clinical interest, but indepth analysis of imaging method comparison can only be achieved in a large scale prospective multicentric study, which shall be initiated as consequence of this report. Moreover, our database did not suffice to include MRI as a second reference method and to include a larger set of patients with multiple or morphological complex cysts. Both EUS and MRI have distinct advantages and limitations in the CPL scenario^29,30^ and must be considered for future trial design. It should also be noted that the examiners for TAUS and EUS were not consistently blinded to the results of the alternative imaging method. However, this is of limited significance when considering the potential role of TAUS in future follow-up scenarios where TAUS will succeed the index exam by the reference method.

In conclusion, the present study sheds new light on the potential role of TAUS as an alternative and cost-effective method for monitoring selected patients with CPL, which could simplify follow-up protocols in the future. Nevertheless, due to the limitations of the study and the suboptimal sensitivity of TAUS in detecting morphological irregularities, despite the use of a patient selection scheme, there is an urgent need for a prospective, multicenter, bi- or trimodal study to finally evaluate the utility and limitations of TAUS as an alternative examination method.

## Methods

### Study design

We conducted a monocentric retrospective analysis at Leipzig University Hospital, including adult patients (≥ 18 years) with CPL who underwent both endosonographic and transcutaneous ultrasound examinations within a period of 6 months between January 2016 and June 2022. This period was determined based on an estimated annual case volume of approximately 20 cases per year. Imaging and clinical data (a.o. BMI, age, sex) were retrieved from the hospital’s picture archiving system (ViewPoint versions 5 and 6, GE HealthCare) and clinical data system (SAP Industry Solution Healthcare – IS-H).

Patients were excluded from the analysis if one of the following criteria was met: previous surgical interventions on the pancreaticobiliary system altering the applicability of EUS and/or TAUS, acute pancreatitis or an acute episode of chronic pancreatitis within 6 months prior to ultrasound examinations, or the presence of underlying malignant disease within the last five years prior to study examinations.

The patients were stratified according to the detectability of CPL on TAUS and the presence of suspicious features of the lesion (Fig. 1).

### Ethics and registration

The study was conducted in accordance with the Declaration of Helsinki. The protocol was approved by the Ethics Committee of the Medical Faculty of the University of Leipzig (407/22-EK) and registered at the German Registry of Clinical Studies (DRKS, register number DRKS00030609). Given the retrospective nature of the analysis, the Ethics Committee of the Medical Faculty of the University of Leipzig (407/22-EK) waived the requirement for individual informed consent.

### Endoscopic ultrasound (EUS) and transabdominal ultrasound (TAUS) procedures

All EUS examinations were performed by board-certified gastroenterologists with more than three years of endoscopic training at the hospital’s interdisciplinary endoscopy unit (> 9000 gastrointestinal endoscopies per year). Patients underwent EUS in propofol-induced sedation using radial and/or linear EUS scanners according to examiner preference (PENTAX EG 367, EG 3870UTK, Fujinon EG 580UR, EG 580UT). The institution’s protocol for EUS examination of CPL comprises transduodenal and transgastric evaluation of the pancreas from defined scope positions and includes a detailed description of the pancreatic morphology^31^. CPL was defined as a focal lesion in the pancreatic parenchyma exhibiting relevant anechoic areas. For any detectable CPL, the maximal diameter, localization, morphology, and pancreatic ductal system (dilatation, calibre changes, CPL connection) were recorded according to the clinical standard (assessment of suspicious morphology: size, echogenicity, pancreatic duct diameter, cyst wall appearance and vascularization, presence of septa and / or mural nodules).

All TAUS examinations were performed by trained (according to the DEGUM level I or higher^32^) and experienced (more than 800 examinations) examiners at the hospital’s interdisciplinary ultrasound unit (> 10,000 abdominal procedures per year). The evaluation of the pancreas was performed in the supine position and, if necessary, in the left lateral or upright position. The examinations were performed using high-end ultrasound systems (Canon Aplio series, GE Logic e9 series)^21^. The standard examination protocol comprises the evaluation of the pancreas in the epigastric sagittal and transverse planes as well as by a lateral translienal approach^27^. The description of pancreas morphology and CPL characteristics was consistent with that of the EUS protocol.

Additionally the EUS and TAUS images and reports were reviewed by a group of trained endoscopists and ultrasound experts at the daily case presentation of the Division of Gastroenterology.

### Morphological assessment of CPL and definition of cross-method agreement

The morphological evaluation of CPL via EUS and TAUS applied the following criteria: size, location, echogenicity, delineation, and vascularization (using Doppler techniques). In patients with more than one CPL, the largest lesion was assessed.

The criteria for differentiating between nonsuspicious and suspicious CPL were as follows: an echogenic lesion, an irregular boundary, a connection to the pancreatic main duct, pancreatic duct dilatation > 5 mm, a vascularized cyst wall, a thickened cyst wall, solid parts, or mural nodules. The criteria selected were based on the European and international evidence-based guidelines for cystic pancreatic neoplasms^1,10^. If any of these criteria were met, the lesion was rated as “suspicious” by either method. In addition, all CPL classified as “suspicious” in the original examination report were deemed suspicious irrespective of further documentation of the CPL morphology. If the CPL was classified identically in both modalities, cross-method agreement was recorded.

### Statistical analysis

All the statistical analyses were performed using Graph Pad Prism version 10. The Shapiro‒Wilk test was used to assess the normality of the distribution of the variables. Statistical differences between defined groups were evaluated based on parametric or nonparametric tests according to the presence or absence of a normal distribution. The Mann‒Whitney U test was used for the analyses of noncategorical parameters, and Fisher’s exact test was used for categorical parameters. The Wilcoxon matched-pairs signed-rank test was applied to examine nonparametric matched variables. Statistical outlier correction was performed with the Graph Pad Prism specific robust regression and outlier removal (ROUT) method with a Q of 1%. The Pearson correlation coefficient (r) was calculated to test the correlation of variables.

Univariate and multiple logistic regression analyses were performed to investigate the impact of different variables on the detection of pancreatic cystic lesions via TAUS. The primary measure for the predictive performance of the logistic regression model was the area under the curve (AUC) of the receiver operating characteristic (ROC) curve. The models generated by these analyses were evaluated using the Akaike information criterion (AIC).

As the analysis was an exploratory post hoc analysis, all p values were interpreted descriptively. No adjustment for multiple testing was adopted. All the statistical tests used an α-level of 0.05, and statistical significance was defined as *p* > 0.05 (ns), *p* < 0.05 (*), *p* < 0.01 (**) or *p* < 0.001 (***).

## Electronic supplementary material

Below is the link to the electronic supplementary material.

Supplementary Material 1

Supplementary Material 2

Supplementary Material 3

## Data Availability

The dataset analysed during the current study is included as supplementary information and labelled data_TrueControl_Scientific-reports.xlsx. Further specific patient data is available from the corresponding authors upon reasonable request.
