# Supplementary figures and images for: Transabdominal ultrasound for the characterization and follow-up of cystic pancreatic lesions
Source: Sci Rep. 2025 Jun 20;15:20228. doi: 10.1038/s41598-025-07136-w (PMC12181248; doi:10.1038/s41598-025-07136-w)

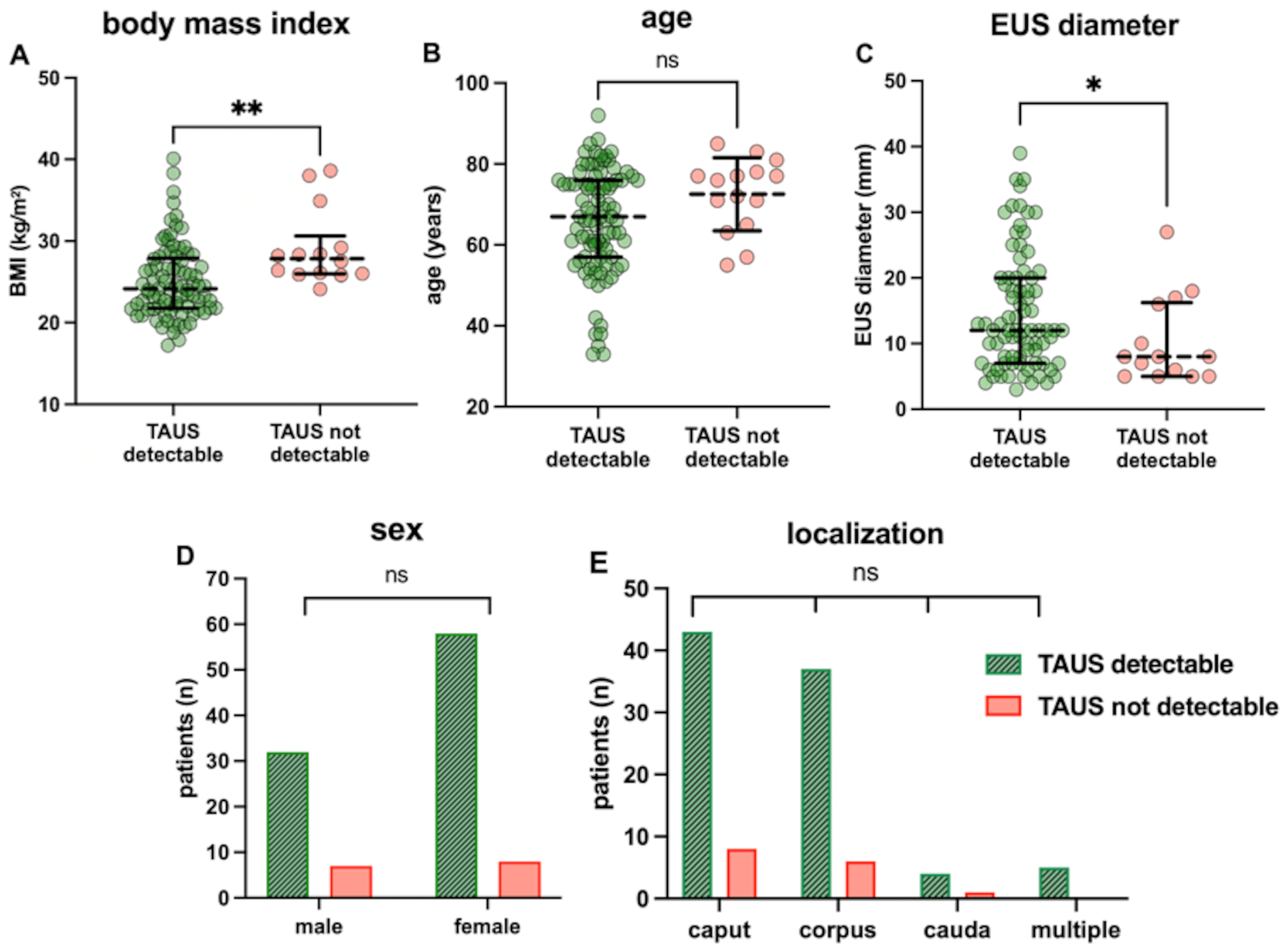

Supplement: Supplementary file 1 — Supplementary Material 1 [file 41598_2025_7136_MOESM1_ESM.jpg]
