# Supplementary material for: Transabdominal ultrasound for the characterization and follow-up of cystic pancreatic lesions
Source: Sci Rep. 2025 Jun 20;15:20228. doi: 10.1038/s41598-025-07136-w (PMC12181248; doi:10.1038/s41598-025-07136-w)
Supplement: Supplementary file 3 — Supplementary Material 3 [file 41598_2025_7136_MOESM3_ESM.docx]

**Supplemental Figure 1.** Comparison of the body mass index (BMI, **A**), age (**B**) and sex (**D**) of the patients with cystic pancreatic lesions (CPL) which are “TAUS detectable” (n = 90, CPL detectable via endoscopic endosonography; EUS and transcutaneous ultrasound; TAUS) and “TAUS not detectable” (n = 15, CPL only detectable via EUS) as well as the localization (**E**) and determined EUS diameter (**C**) of the CPL. The groups are presented as medians and interquartile ranges. All the statistical tests used an α-level of 0.05, and statistical significance was defined as p > 0.05 (ns), p < 0.05 (*), p < 0.01 (**) or p < 0.001 (***).
